# Chronic Disease Prevention and Control: Coming of Age at the Centers for Disease Control and Prevention

**Published:** 2009-06-15

**Authors:** Janet L. Collins, Jeffrey P. Koplan, James S. Marks

**Affiliations:** National Center for Chronic Disease Prevention and Health Promotion, Centers for Disease Control and Prevention; Emory University, Atlanta, Georgia; The Robert Wood Johnson Foundation, Princeton, New Jersey

The Centers for Disease Control and Prevention's (CDC's) National Center for Chronic Disease Prevention and Health Promotion (NCCDPHP) has entered its 20th year — at the crossroads between adolescence and adulthood. As the 3 directors of the center to date, we offer our perspective on its developmental path and on the opportunities and challenges that lie ahead.

Former CDC Director William Roper described public health as the "intersection of science and politics" (1). This description speaks volumes about the strategies we employ and the resources we have. Spurred by the 2008 presidential election, current national debate includes renewed interest in health care reform. Reform discussions largely revolve around alternative mechanisms and financing needed to achieve universal coverage for medical care. Too often absent in these discussions is the critical need for population-based prevention to protect health in the first place. "Health in all policies and settings" could be a unifying strategy to complement the delivery of clinical preventive services and care. This expanded vision of health reform will depend on a robust public health system that can address the leading determinants of health and health care cost: chronic disease prevention and control.

The backbone of a strong public health system is the national, state, and local public health infrastructure. Twenty years have brought growth to core chronic disease prevention and control programs at each of these levels, but the programs remain weak and fragmented and are of secondary importance in too many public health departments. The good news is that all 50 states and the District of Columbia have programs that focus on tobacco use, diabetes, breast and cervical cancer screening, comprehensive cancer control, and the Behavioral Risk Factor Surveillance System (BRFSS). These programs succeed by monitoring health risks, reducing tobacco use and exposure to secondhand smoke, detecting early disease, and improving the length and quality of life of people living with diabetes and cancer. Effective state programs reflect a complementary relationship with CDC, in which CDC provides technical and financial support, and state chronic disease programs innovate, test, and share experiences that move the field forward.

In contrast, programs that promote improved nutrition and increased physical activity are inadequate. Although increasing obesity rates have raised nationwide concern in the private and public sectors and among children's advocates, only half of the states have federal resources to fund public health activities in this area. Loss of funds and declining purchasing power since 2001 have effectively removed more than $100 million from CDC resources devoted to this issue. Because states must compete for these limited funds, every 5 years programs are built up in some locations and dismantled in others, leaving millions unserved as the result of small distinctions between high-quality proposals. The same situation applies to programs that address heart disease, stroke, arthritis, and oral health. Although the past 20 years have brought a deeper and stronger scientific basis for public health approaches to chronic disease prevention, public health remains without the basic resources it needs to establish strong chronic disease prevention programs at the state and local levels.

Despite the challenges that patchwork funding presents, national advances in chronic disease prevention programs have been profound. One such advance was the 2006 establishment of CDC's Division for Heart Disease and Stroke Prevention to address the nation's first and third leading causes of death. This new organizational unit, coupled with the existing Division of Cancer Prevention and Control, means that at a national level, public health is tackling the nation's biggest killers. *A Public Health Action Plan to Prevent Heart Disease and Stroke* — and the public and private partnership created by it — charts the course to achieve national goals for preventing heart disease and stroke through 2020 and beyond. For the first time, an action-oriented, national public health plan has been developed for the leading cause of death in the country.

In cancer control, 20 years have seen the maturation of the National Breast and Cervical Cancer Early Detection (B&C) Program. Publicly funded screening services can be provided at a cost consistent with screening in the general population (2). Since 1991, more than 3.3 million women have been screened through the B&C Program (3), and thousands of cancers have been detected at treatable stages (4). The program has reached at-risk women who have historically been missed by screening programs (5). Unfortunately, current resources limit public health authorities to reaching just 15% to 20% of women who are eligible for mammography services.

CDC's National Comprehensive Cancer Control Program, established in 1998, is an innovative systems approach to state, tribal, and territorial planning and program delivery in cancer control. As a result of this program, every state, the District of Columbia, 7 tribes/tribal organizations, 6 US Pacific Island jurisdictions, and Puerto Rico have a comprehensive cancer control plan and an active cancer coalition that brings together expertise and capabilities to address cancer prevention, control, and survivorship. Through this program, many states are highlighting the importance of colorectal cancer screening, which could prevent 70% to 90% of deaths from colorectal cancer if all precancerous polyps were identified and removed (6). The recent action of Congress to add $25 million to CDC's fiscal year 2009 budget for colorectal cancer will allow CDC to begin to establish a nationwide screening program that covers screening and diagnostic follow-up care to low-income men and women with no or inadequate health insurance coverage for these services.

Twenty years have also brought scientific findings that have expanded the ability of the public health community to take action. A key contributor is the CDC-supported network of 33 Prevention Research Centers (PRCs), which collaborate with community, academic, and public health partners to conduct participatory research and to put that research into practice. This network is CDC's largest extramural research program and has helped put community-based participatory research on the national map. Each center has a community advisory committee that considers the community's perspective in light of scientific evidence. A good example of this partnership is the way that researchers at the University of Washington's PRC worked with seniors to develop Enhance Fitness, recognized by the National Council on Aging as one of the top 10 physical activity programs in the United States. Evaluation results demonstrated improved outcomes in physical functioning, enhanced socialization, decreased depression, decreased physical pain, and reduced health care costs (7). In 8 years, Enhance Fitness progressed from 1 site to more than 300 sites in 26 states, reflecting the PRC network's emphasis on committed, long-term partnerships to develop, translate, and disseminate effective programs.

A noteworthy piece of scientific work comes from the National Institutes of Health-funded Diabetes Prevention Program (DPP), a clinical trial aimed at discovering whether diet and exercise or the oral diabetes drug metformin could prevent or delay the onset of type 2 diabetes (8). DPP results show that type 2 diabetes can be prevented or delayed with moderate weight loss and improvements to nutrition and physical activity behaviors. Unfortunately, data from this well-controlled, well-resourced clinical trial have yet to be translated into widespread public health practice. CDC is conducting several pilot programs to identify people who are at high risk for type 2 diabetes and enroll them in diabetes prevention interventions based on the DPP. Early results suggest that outcomes similar to that of the original trial can be achieved at a fraction of the cost.

In addition to applied research, evaluation and surveillance are pivotal to NCCDPHP's public health achievements. A prime example is the BRFSS. BRFSS designers recognized not only the importance of state data in influencing and evaluating the success of public health programs but also the need to tackle multiple public health issues in a single surveillance system. With the BRFSS's built-in flexibility, states can add questions of high salience. Moreover, innovative modules have opened up major areas of public health action, such as work in mental health, quality of life, and experiences of racism and its relationship to health outcomes. Perhaps most noteworthy in the past decade is the use of BRFSS data to document rising obesity rates and to drive public attention and public health response to this epidemic. Recent advances, such as the introduction of SMART (Selected Metropolitan/Micropolitan Area Risk Trends) BRFSS, which provides data for hundreds of counties by summing across multiple years to make stable estimates at the local level, demonstrate that BRFSS is an evolving, world-class data system (9).

A major structural change for the center occurred in 2006 with the transfer of the Office of Public Health Genomics from CDC's Office of the Director to NCCDPHP. This office continues its work to establish public health genomics as a multidisciplinary field concerned with the effective and responsible translation of genome-based knowledge and technologies to improve population health. The Office of Public Health Genomics has led the way in using genomics-based health applications, promoting family history as a tool for disease prevention, and examining, through CDC's National Health and Nutrition Examination Survey, the prevalence and association of 90 genetic variants with specific disease outcomes.

Several other accomplishments are worth noting in this 20th anniversary year. One is the  achievements of the youth media campaign VERB. In his last year in office, Congressman John Porter from Chicago called for the use of paid media to market health to children. NCCDPHP embarked on one of the most innovative projects in its history. Funds at a level unheard of in public health (averaging approximately $70 million per year) were provided to use the same advertising strategies that were employed by the best marketers of children's products. By the end of the 5-year campaign, VERB had won more than 50 major industry awards. More importantly, this campaign, which was "by and for kids," achieved a 75% recognition rate among the target audience (9- to 13-year-olds). As the ultimate measure of success, children who were aware of VERB reported engaging in significantly more physical activity than did children who were unaware of VERB (10). This story ends with disappointment in terms of sustaining meaningful changes in the health of youth. Despite evidence of nationwide effectiveness, VERB funding was halted at the end of 5 years.

Another area of transformation is CDC's work with communities, including programs that show success in eliminating racial and ethnic health disparities. Communities that participate in the Racial and Ethnic Approaches to Community Health (REACH) program are innovators in strategy and intervention. Their documented successes in reducing and eliminating health disparities speak powerfully to the importance of engaging local leaders and organizations, forging strong community partnerships, and recognizing cultural influences and historical legacies (11). The Healthy Communities Program (which builds on the Steps Program established in 2003) simultaneously addresses chronic diseases such as obesity, diabetes, heart disease, physical inactivity, poor nutrition, and tobacco use by creating a groundswell of activity in local communities, through schools, worksites, health care settings, and other community institutions. The Healthy Communities Program emphasizes public health interventions that are evidence-based and that reach beyond public health to community health by bringing together business, transportation, and city planning sectors. The WISEWOMAN (Well-Integrated Screening and Evaluation for Women Across the Nation) program serves women aged 50 to 64 years and builds on the B&C Program's extensive outreach to uninsured and underinsured women who are at or below 250% of the federal poverty threshold. The community-based WISEWOMAN programs provide standard preventive services, including blood pressure and cholesterol testing, as well as lifestyle programs that promote good nutrition, physical activity, and smoking cessation.

CDC and partners such as the YMCA of the USA, the National Association of County and City Health Officials, the National Recreation and Park Association, the National Association of Chronic Disease Directors, and the Society for Public Health Education are working to share lessons learned from REACH, Healthy Communities, Steps, and WISEWOMAN through carefully developed tools and training. Moving these demonstrations into widespread practice will require political will at the national, state, and local levels to provide resources that enable local communities to take action. As an example of such political will, the Minnesota state legislature recently voted to invest $47 million in 2 years to establish a new statewide health improvement plan — owing to the success of the state's Steps Program in promoting health at the community level.

Our work in maternal, child, and adolescent health is also reaching new heights. Together, the Division of Reproductive Health (DRH) and the Division of Adolescent and School Health (DASH) have reduced the rate of unintended teen pregnancy. DRH, through its Safe Motherhood program, tackles a wide range of maternal and child health issues, including infertility, premature birth, gestational diabetes, tobacco use during pregnancy, and postpartum depression. DASH is the nation's "go-to" location for resources and assistance to build healthy youth and healthy schools. DASH's direct involvement with the nation's state and local education agencies and organizations (in concert with traditional public health agencies) has enabled work with the nation's schools and is a model for work with other sectors.

Advances in the areas of tobacco, nutrition, physical activity, and alcohol represent some of the most important work of the center. Tobacco control has set a new and powerful paradigm for prevention by documenting the influence of policy and media. We see signs of the new paradigm being applied to nutrition through innovative local and state initiatives to influence food choices in day care centers, schools, and hospitals; restrict fast food and liquor store densities; provide calorie information on menu items; improve food labeling practices; limit food advertising to children; provide incentives for full-service grocery stores in urban "food deserts"; and reduce salt in the nation's food supply.

Finally, our work with the CDC Foundation, a 501(c)(3) charity, helps donors and CDC scientists achieve common goals. For example, the Avon-CDC Foundation Mobile Access Program provides mammography screening vans that serve women in geographically remote areas. Services are made possible through a $4.1 million gift from the Avon Foundation. Through such partnerships with the CDC Foundation, NCCDPHP is able to extend its reach and capabilities. Current donor investments to NCCDPHP through the CDC Foundation total $60 million, including a sizeable grant from the Bloomberg Philanthropies to establish global surveillance of adult tobacco use.

Although the major chronic diseases and their risk factors are distinct in terms of biology, prevention, and treatment, they share many similarities. Populations at risk for 1 chronic disease are often at risk for multiple chronic diseases. Common settings, such as schools, worksites, health care organizations, and communities serve as intervention sites for the prevention of multiple risk factors, early detection of disease, and promotion of self-management programs for chronic disease. Lastly, coordinated strategies, such as those involving supportive public policy, social and physical environments, system changes, media, and technology, are required to address nearly all chronic disease risk factors and conditions.

Recognizing the necessity for improved program integration, NCCDPHP is working with states and communities to develop and evaluate new models for chronic disease prevention that focus on populations rather than on risk factors and diseases. Four states — Colorado, North Carolina, Massachusetts, and Wisconsin — have established unified work plans that preserve the integrity of Congressional funding lines but do so in the context of a comprehensive plan. These models are precursors to a new way of doing business that maintains focus on evidence-based best practices while maximizing the impact of investments across categorical programs.

We have described the substantial advances that NCCDPHP has made in chronic disease prevention and control and the potential — and need — for future development. Public health has played a central role in many of the greatest health achievements of our times and is positioned to achieve much more (12). However, we believe that the greatest challenge to public health is solving the investment problems that have plagued chronic disease prevention and control for too long. We conclude with 4 recommendations for the center and its work.

- Prevention parity. Preventive actions to maintain health in the absence of disease are underused and undervalued. NCCDPHP and its partners must be the outspoken leaders on behalf of prevention, its financing, and its delivery. Prevention methods are required to demonstrate cost-effectiveness, if not cost savings, before they are employed. At the same time, costly, medical procedures and treatments are not held to the same standards. The nation needs 1) a level playing field for the assessment of both preventive and therapeutic interventions and 2) support for interventions that improve health at a reasonable cost. Public health systems research, cost-effectiveness research, and translation research are all needed to advance and support the prevention mission.
- Optimal health for all. High-coverage, long-lasting, and low-cost strategies, such as laws for clean indoor air and water fluoridation, are the hallmarks of effective public health practice. Constant vigilance is required, alongside these efforts, to ensure that we are reaching populations that face the greatest inequities in health. Intensive community efforts focused on achieving health equity, such as those demonstrated by REACH, also will be critical to success.
- Health in all policies and settings. Increasing health care costs and subpar health outcomes are illuminating the importance of prevention. Even the broader health sector cannot deliver optimal health outcomes on its own. Policies and practices in education, housing, transportation, and agriculture have far-reaching health effects but are not engaged or evaluated for those outcomes. Work in the area of social determinants of health highlights the importance of environmental, social, political, and economic conditions on health. Given the influence of multiple sectors on health, the Department of Health and Human Services' *Healthy People 2020* health objectives for the nation will have the best chance of success if they explicitly call for the engagement of key sectors and if the objectives are adopted and addressed by the president's full cabinet.
- Worldwide engagement. The changing landscape of global disease patterns — from infectious to noninfectious causes — will require more leadership and a more global engagement from NCCDPHP than ever before. NCCDPHP's global surveillance activities establish a foundation for this work by providing public health data in more than 150 countries. CDC's bilateral and multinational work on social determinants of health, health promotion, and tobacco control informs progress in chronic disease prevention and control in the United States and abroad. These efforts should be leveraged and expanded.

We are proud to have been part of the growth of NCCDPHP and to have participated in its support of the remarkable work of state and local health departments, partners, and colleagues in the past 2 decades. What began as a relatively new frontier in public health is now accepted as a centerpiece for health and wellness in the country. Ultimately, matching the intensity and reach of our prevention efforts to the scope of the chronic disease challenges will be necessary to deliver on the promise of optimal health for all.
